# HPV genotypes in paraffin sections of non-cervical squamous cell carcinoma in Qingdao of China

**DOI:** 10.3892/ol.2013.1181

**Published:** 2013-02-06

**Authors:** ZHI DI GAO, QI PAN, HONG LV, YANG SUN, XIAOYE MA, ZUORONG QIN, YU PING SUN

**Affiliations:** 1 Institute of Medicine, Shandong University, Jinan 250012;; 2 Division of Oncology, Qingdao Hiser Medical Group, Qingdao 266033;; 3 Departments of General Surgery, Qingdao Hiser Medical Group, Qingdao 266033;; 4 Pathology, Qingdao Hiser Medical Group, Qingdao 266033;; 5 Otorhinolaryngology, Qingdao Hiser Medical Group, Qingdao 266033;; 6 Department of Tumor Treatment, Jinan Central Hospital, Institute of Medicine, Shandong University, Jinan, Shandong 250012, P.R. China

**Keywords:** squamous cell carcinomas, human papillomavirus genotype, polymerase chain reaction

## Abstract

Human papillomaviruses (HPVs) are the cause of cervical cancer and possibly a subset of squamous cell carcinomas (SCCs) in other sites. However, the prevalence and distribution of HPV subtypes remain unclear. In the present study, we collected and analyzed 511 paraffin sections of non-cervical SCC from patients in Qingdao, China, for the presence of HPV using polymerase chain reaction (PCR). We identified that 55.77% (285/511) of the samples were positive for HPV infection. There was a significant association between HPV type and the different sites of SCC. An association between HPV-positive cases and tobacco, alcohol, age and tumor differentiation was demonstrated. The information provided by this study may be important for further investigation into the association between HPV and SCC. High-risk HPV subtypes were associated with the malignant degree of SCC. This study provided a theoretical basis for the preventative treatment of non-cervical SCC using HPV vaccines.

## Introduction

Human papillomavirus (HPV) infection has been globally reported as being involved in tumors in several types of cancer, including genital mutilation cancer, penis cancer, lung cancer, head and neck tumors, gastric cancer, breast cancer, colon cancer, skin cancer and esophageal cancer. Numerous studies have shown that HPV infection is closely related to squamous cell carcinoma (SCC) and it has been determined as an important factor in the induction of cervical SCC. Preliminary studies have been carried out to determine whether HPV exists in SCCs in non-cervical sites (1). Studies have been carried out in various geographical locations, therefore, sample volumes, means of detection, virus types and the distribution of HPV subtypes are often detected in SCCs from different sites. Few studies have characterized the distribution of the specific subtypes of HPV in the varying grades of SCCs from different sites.

This study aimed to explore the correlation between the different subtypes of HPV and the different sites of non- cervical SCCs in 511 patients from Qingdao, China, using a polymerase chain reaction (PCR) detection method. The high prevalence of HPV6/16 and the lack of HPV18 in esophageal SCC and lung SCC may point to specific virus-tissue interactions.

## Materials and methods

### 

#### Specimen selection

Surgical resection specimens (n=511) were retrieved from Qingdao Hiser Medical Group and Qingdao Center Hospital between 2006 and 2011. All patients belonged to low socioeconomic strata and the majority of these were agricultural workers. None of the cases had been treated with radio- or chemotherapy prior to surgery. The specimens consisted of 27 tongue SCC, 79 nasopharyngeal SCC, 196 lung SCC, 185 esophageal SCC and 24 rectal SCC cases. The specimens for the control group, which were cut from the margins of the tissues in the same cases, were confirmed as non-tumor tissue by pathology. There were 397 specimens from males, while 114 were from females. According to Broders’ classification, the histopathological staging of all SCC cases were as follows: 238 stage I, 234 stage II, 30 stage III and 9 stage IV cases. Their ages ranged from 26–81 years (mean, 63 years). None of the cases had distant metastasis. All specimens underwent a regular dewaxing process and were cut continuously into 4-μm-thick sections for HPV testing. The histology from all cases was reviewed by two pathologists who confirmed the diagnosis. Discrepant cases were resolved by histological evaluation by a third pathologist.

### HPV testing

#### HPV genotyping test kit

DNA was extracted from each of the 511 paraffin-embedded specimens using the HPV genotyping test kit according to the manufacturer’s instructions (Asian Research Centre of Molecular Diagnostic, Co., Ltd., Shenzhen, China). The kit applied DNA-chip technology based on *in vitro* amplification combined with PCR reverse dot blotting.

The kit used special primers to obtain 23 types of HPV amplification products by PCR. It was hybridized with probes, which had 5 low-risk genotypes and 17 high-risk genotypes fixed in the membrane. It determined the HPV genotype by its hybridization signal.

#### HPV DNA extraction

Exfoliated SCC cells were collected and added to 50 μl lysate and centrifuged at 13,000 rpm for 10 min after a boiling water bath for 10 min. The supernatant was reserved for template DNA.

#### PCR amplification

Template DNA (5 μl) was added to the PCR mix. PCR conditions were as follows: 50°C for 15 min, denaturation at 95°C for 10 min followed by 40 cycles of 94°C for 30 sec, 42°C for 1 min and 30 sec, 72°C for 30 sec and 72°C for 6 min.

#### Hybridization

The PCR product and membrane were incubated for ≥1 h and 30 min in a hybridization incubator and then kept in a boiling water bath for 10 min in 5 ml solution A. The DNA became purified after this step.

#### Membrane wash

The membranes were washed in a hybridization incubator for 5 min in solution B at 51°C to remove proteins and other contaminants.

#### Color agent

After washing the membrane, it was incubated for 30 min in solution A (2X SSC, 0.1% SDS) mixed with POD (Solution A:POD = 2000:1). It was then colored for at least 30 min in color liquid, which was added to 19 ml solution C (1M 100 ml sodium citrate), 1 ml TMB and 10 μl 3% H_2_O_2_. The color liquid was removed and deionized water was added. The HPV genotype was determined by its hybridization signal.

The film articles were placed on the reading instrument scanner and the results were saved. The wet film article was placed into an airtight seal in a hermetic sealing bag and stored at 2–8°C in a refrigerator for preservation.

#### Statistical analysis

Statistical analysis was performed using Fisher’s exact test and Student’s t-test. P<0.05 was considered to indicate a statistically significant difference.

## Results

### HPV infection in various sites of SCC

**Figure 1:**
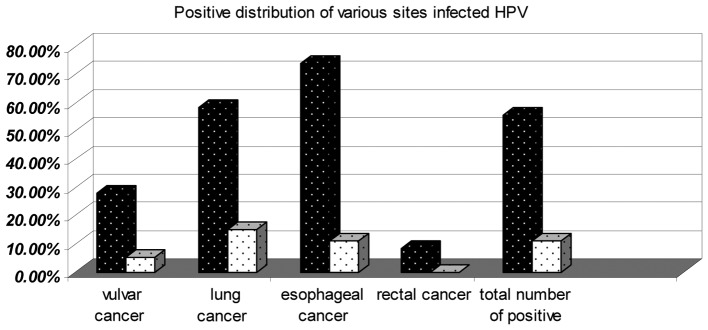
Positive distribution of various sites infected with HPV. HPV, human papillomavirus.

Overall, HPV was detected in 285 of 511 (55.77%) SCC tissues compared with 55 of the 511 (10.76%) normal tissues. The HPV-positive distribution for the tissue from each SSC site was higher than for the normal tissue at its corresponding site. The HPV-positive distribution in 137 of 185 (74.05%) esophageal SCCs and 114 of 196 (58.16%) lung SCCs was higher than for the other SCC sites (Fig. 1).

### Distribution of HPV subtypes in various sites of SCC

**Figure 2:**
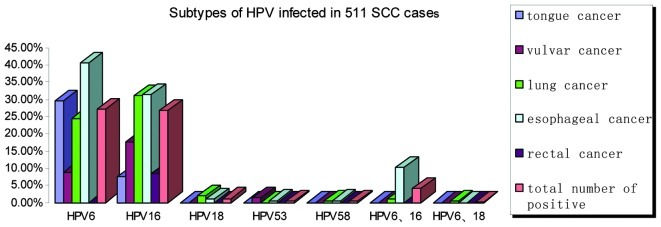
Subtypes of HPV infection in 511 SCC cases. HPV, human papillomavirus; SCC, squamous cell carcinoma.

Five HPV genotypes were identified, including HPV6, 16, 18, 53 and 58. High-risk HPV was composed of HPV16, 18, 53 and 58, whereas low-risk HPV was HPV6 only. Overall, 147 of 285 (51.58%) cases were positive for high-risk HPV, of which 137 (48.07%) were associated with HPV16, 6 (2.11%) with HPV18, 2 (0.70%) with HPV53 and 2 (0.70%) with HPV58. The number of cases with low-risk HPV positivity (138/285, 48.42%) was higher compared with other HPV types and the majority of cases had esophageal cancer. HPV6 and HPV16 comprised a large proportion of the HPV-infected cases (Fig. 2).

### Patients with HPV-positive factors in SCC

**Figure 3:**
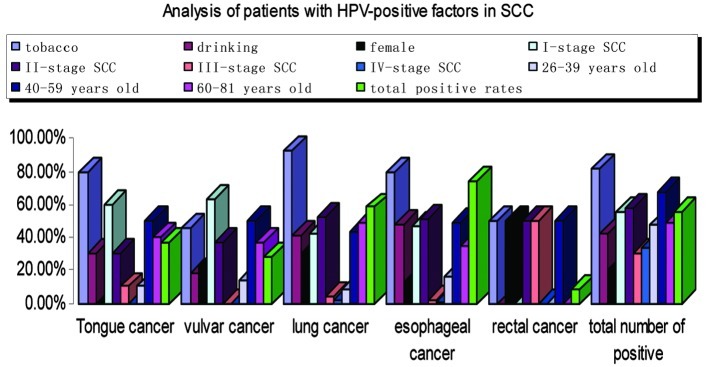
Analysis of patients with HPV-positive factors in SCC. HPV, human papillomavirus; SCC, squamous cell carcinoma.

Follow-up information was available for 285 HPV-positive patients who were diagnosed with SCC stages I-IV (Fig. 3), including 129 (45.26%) with stage I, 92 (32.28%) with stage II, 53 (18.60%) with stage III and 11 (3.86%) with stage IV. Tobacco (82.11%), drinking (42.11%) and being middle-aged (67.17%) were important factors, however tobacco was the most important (Fig. 3).

### Association between subtypes of HPV infection and the stage of SCC

**Figure 4:**
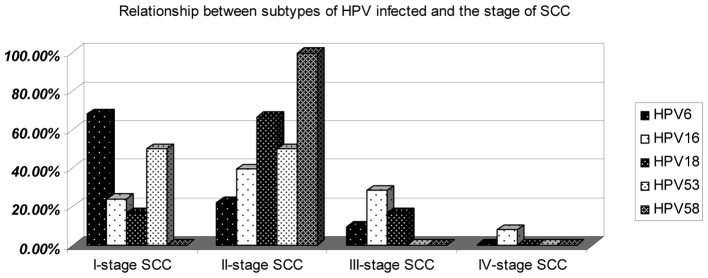
Association between subtypes of HPV infection and the stage of SCC. HPV, human papillomavirus; SCC, squamous cell carcinoma.

There were 137 HPV16-infected cases, of which 33 (24.09%) were in stage I, 54 (39.42%) in stage II, 39 (28.47%) in stage III and 11 (8.03%) in stage IV. For HPV6-infected cases, there were 94 (68.12%) in stage I, 31 (22.46%) in stage II, 13 (9.42%) in stage III and 0 (0%) in stage IV. There was 1 (16.67%) case in stage I, 4 (66.67%) in stage II and 1 (16.67%) in stage III for HPV18-infected cases. There were only two cases infected by HPV53, 1 of which was in stage I and the second was in stage II. Only two cases were infected by HPV58 and these were in stage II (Fig. 4).

## Discussion

Cancer incidence in the Qingdao area has increased rapidly, particularly tongue, nasopharyngeal, lung, esophageal and colorectal cancer. These five types cancers are in the top ten most commonly diagnosed tumors. Of the top ten, squamous cell carcinomas are the most commonly observed types of tumor. Studies investigating the correlation between non-cervical SCC and different subtypes of HPV infection remain limited, despite their increasing incidence in the last 20 years. Studies have reported that HPV-positive tumors have a better response to therapy (2). Numerous studies indicate that a possible use of HPV diagnosis is as an additional aid for survival prognosis, at least for localizations such as tonsils or for females rather than males, where an improved clinical outcome has been demonstrated (3–5). However, there have been no further studies with regard to the specific type of positive HPV which is useful for determining the prognosis of SCC. Whether the high- or low-risk HPV type is better for accurate prognosis remains unclear. HPV vaccines have been used in numerous areas as one of the effective measures to prevent cervical SCC. Whether it is possible to administer them as a conventional measure to prevent SCC in other sites is unknown. A previous study showed that the subtypes of HPV infection presented with a regional distribution (6). The main factors which affect the correlation between HPV infection and non-cervical SCC in Qingdao and the correlation between these factors and the cases of SCC with HPV infections have yet to be explored. Therefore, we conducted the present study in Qingdao, China.

In the present study, we collected and analyzed 511 non-cervical SCC specimens for the presence of HPV infection using PCR and non-isotopic *in situ* hybridization. In the noncervical SCC samples, 19.30% (285/511) were positive for HPV detection, while 10.76% (55/285) of the control samples were positive. The presence of HPV infection was significant in noncervical SCC cases (P<0.05). HPVs have been categorized by their genotype into low- and high-risk types, according to the risk of the virus causing SCC of the uterine cervix (7). In this study, we hypothesized that HPV infection is closely correlated with the high- and low-risk types of SCC. In 2007, the American Cancer Society announced the use of the HPV vaccine to prevent cervical SCC in females (8). The quadrivalent vaccine for HPV types 6/11/16/18 was successful (9). This study revealed a significant positive rate of HPV6 and HPV16 in cervical SCC patients, which indicates that it may be possible to administer the HPV vaccine for the prevention and treatment of SCC.

HPV invades basal cells through small amounts of epithelial damage. The virus particle binds with receptors on the cell surface, enters into the cell and transfers to the nucleus, where the released virus gene is replicated, resulting in chromosomal changes of the host cell. The cell with DNA damage may be the foundation for the induction of cancer (10). Studies have shown that high- and low-risk types of HPV infection are present in a variety of the SCC subtypes, including HPV16, 18 and 6 (11,12). However, whether HPV infection was correlated with epithelial tumor differentiation was not determined. The total positive rate of the high-risk type was 51.58%, of which 48.07% of cases were HPV16-infected. The positive rate of HPV16 infection was 100% in stage IV SCC, 66.67% in stage III, 58.70% in stage II and 25.58% in stage I. The total positive rate of the low-risk type was 48.42% and this consisted of the HPV6 subtype only. In stage IV SCC, the positive rate of HPV6 infection was 0.00%, while there were 24.53% HPV6-positive cases in stage III, 33.70% in stage II and 72.87% in stage I. The distribution in all stages of non-cervical SCC between high- and low-risk types was clear; low-risk types were mainly distributed in the higher grades of differentiation and high-risk types were mainly distributed in the lower-level differentiated SCCs. This is in agreement with numerous studies which show high expression rate of high risk HPV infection in SCC patients (6,13). Statistical analysis demonstrated that the high-risk HPV subtype was the most important factor that was proportional to the malignant degree of SCC.

Infection of the uterine cervix with any HPV genotype is associated with high-risk sexual behavior, particularly if started at a younger age. Persistent infection of the uterine cervix with high-risk HPV genotypes, particularly HPV16 and HPV18, is essential for the development of SCC (14). This study showed that tobacco (234/285) was more important than three other factors which were also associated with HPV infection in SCC; drinking, age and gender (P<0.05). The positive rate of HPV infection among middle-aged patients (133/198) was higher than for younger and older ages (P<0.05).

With regard to incidence and prevalence, esophageal cancer exhibits marked geographical variations due to unknown factors between countries, in addition to between different regions of the same country. According to the World Health Organization, incidence rate spectra are located between Western Africa at the low-risk end and China at the high-risk end, including the apparent ‘Asian esophageal cancer belt’ (15). The prevalence of HPV6 (75/137) and HPV16 (58/137) is high in esophageal SCC, while for HPV18 (2/137) it is low. The prevalence of HPV6 (48/114) and HPV16 (61/114) is also high in lung SCC, while HPV18 (4/114) is also low. The two subtypes of HPV infection in esophageal and lung SCC had significant differences when compared with other sites of SCC (P<0.05). The high prevalence of HPV6/16 and the lack of HPV18 in esophageal and lung SCC may point to specific virus-tissue interactions.

This study demonstrated that the high-risk HPV subtype was the most important factor associated with the malignant degree of SCC. The study provided a theoretical basis for the preventative treatment of non-cervical SCC using HPV vaccines. Further study is required to determine the effect of HPV on survival in patients when observed in combination with other prognostic factors.
